# Battery-free fully integrated microfluidic light source for portable lab-on-a-chip applications

**DOI:** 10.1038/s41598-020-69581-z

**Published:** 2020-07-31

**Authors:** Filippo Storti, Silvio Bonfadini, Luigino Criante

**Affiliations:** 1 Center for Nano Science and Technology@PoliMi, Istituto Italiano Di Tecnologia, via Pascoli 70/3, 20133 Milan, Italy; 2 Dipartimento Di Fisica, Politecnico Di Milano, Piazza Leonardo da Vinci 32, 20133 Milan, Italy

**Keywords:** Lasers, LEDs and light sources, Characterization and analytical techniques, Photonic devices, Fluid dynamics, Optics and photonics

## Abstract

Integrating a light source inside a Lab-on-a-Chip (LOC) platform has always been as challenging as much as an appealing task. Besides the manufacturing issues, one of the most limiting aspects is due to the need for an energy source to feed the light emission. A solution independent of external energy sources can be given by Chemiluminescence (CL): a well-known chemical phenomenon in which light emission is achieved because of a chemical reaction. Here we present the fabrication and the characterization of a chemiluminescent light source, fully integrated on a microfluidic platform by means of the direct writing technique known as Femtosecond Laser Micromachining. The key advantage is the possibility to insert within LOC devices light sources with complete placement freedom in 3D, wide flexibility of the emitting source geometry and no external feeding energy. The characterization is carried out by investigating the effect of confining a chemiluminescent rubrene-based reaction in small volumes and the inject pressures impact on the emission spectra. Moreover, exploiting microfluidics principles, it’s possible to move from the typical flash-type CL emission to a prolonged one (several hours). This allows to disengage bulky, external light sources, adding an extra step on the road to real device portability.

## Introduction

LOC devices for biological sample analysis aim to shrink bench-top instrumentation into a single portable platform. The ultimate goal is the integration of one or more functionalities—e.g. sample preparation, reaction/excitation, detection—onto a complete and stand-alone analysis system. The intrinsic advantages of this method have proved to be attractive and particularly suited for the biological and chemical research field. These include: the low sample volume consumption, the faster analysis response time, high sensitivity and portability, due to the reduced device dimensions^1^. Among the broad range of LOC methods, the branch that exploits optical detection techniques is known as optofluidics^2^. This method well fits to biological/chemical sample analysis because of its good sensitivity, due to the improved limit of detection, greater robustness and high throughput. In the last two decades, many studies have presented integrated optical elements to achieve more compact and portable devices^3^. However, the usual working operation of almost all of them is still closely related to the presence of an external power supply. This dependence prevents a more advanced miniaturization and compromises the real portability of the chip. As a result, one of the biggest field challenges—i.e. to reach the independence from external excitation source—is not completely satisfied yet. Furthermore, moving the light source in the same substrate where the microfluidic structures are fabricated leads to an increase of the light probe efficiency by reducing leakages and background noises. For this reason, several interesting solutions have been proposed. Currently, the two most used on-a-chip light sources are dye lasers^4–8^ and organic light-emitting devices (OLEDs)^9, 10^, whose outputs are respectively coherent and incoherent light signals. Other attempts are represented by fluorescent liquid–liquid (${\mathrm{L}}^{2}$)^11^ or liquid–air (LA) waveguides^12^, in which an external laser source promotes fluorescence in a specific region of the microfluidic channel. Then, the incoherent light produced can be guided towards the sample interrogation area, thanks to the refractive index difference of the flowing fluids. Another curious approach is shown by Pagliara et al.^13^ in which the light is emitted by polymeric nanofibers, deposited near the microfluidic channels.

These implementations, while showing the above listed advantages, have all the same critical point: the dependence from an external energy source to feed the emitted light. In fact, for dye lasers, ${\mathrm{L}}^{2}$, LA waveguides and polymeric nanofibers light emission needs to be pumped by another collimated external light source. OLEDs, in the same fashion, can work as emitters only if a voltage is applied. Whereby, to fully satisfy even the portability requirement, innovative light sources are still crucial. In this context, exploiting chemiluminescence properties of some organic compounds could give a significant contribution.

**Figure 1:**
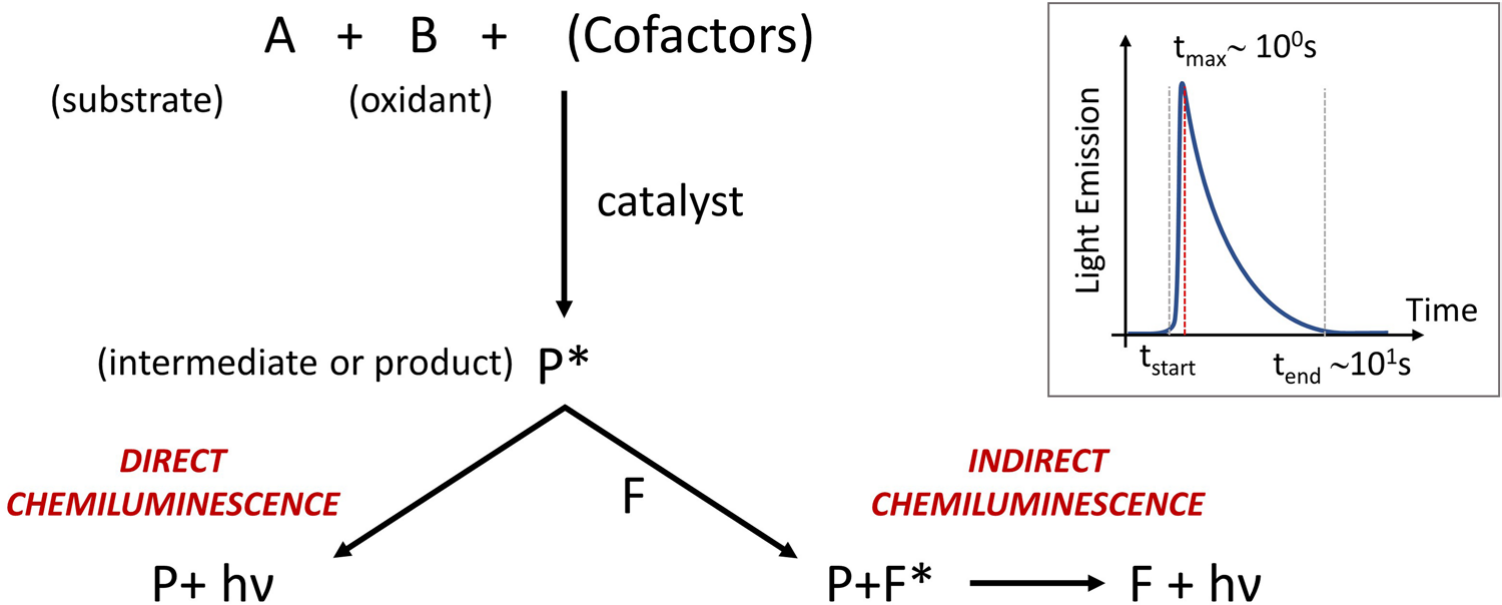
General scheme of a CL reaction mechanism. Two reagents (A and B), with same cofactors, react to produce an electronically excited compound (P*) that will emit*—*directly or indirectly—light. If P* emits itself the CL light the reaction it is known as direct chemiluminescence. Instead when the CL light is emitted by a fluorophore (F) present in the solution as cofactor, thanks to an exchange of energy with P*, the reaction is called indirect chemiluminescence. (Inset) General model of the light emission phenomenon (flash-type) linked with the CL reaction. Emission peak (${t}_{max}$) is reached after few seconds the reaction starting (${t}_{0}$). In most of CL reactions the emission duration (${t}_{end}$–${t}_{0}$) lasts tens of seconds^16^.

CL is a well-known chemical phenomenon in which, as schematically depicted in Fig. 1, two reagents (A and B) produce an electronically excited compound (P*) that will emit—directly or indirectly—light, due to relaxation processes^14, 15^. In our case, the light emission is achieved when the intermediate product P* reacts with a fluorophore (F) present in the solution as cofactor. Then, the fluorophore, excited by the reaction, emits photons through relaxation. This process is known as Indirect Chemiluminescence. Generally, the CL emission reaches an intensity peak after few seconds and the overall emission duration lasts tens of seconds^16^. Thus, this chemical reaction represents a simple, fast and cheap way to produce incoherent light. Furthermore, what makes this mechanism fascinating for LOC application is the typical liquid physical state of the reagents and the absolute independence from external feeding energy sources.

This peculiar “light system” finds its widespread use in detection assays, especially in the biological samples analysis. Specific chemiluminescent compounds are used as a probe, as they emit light once they meet the analytes^17–26^. Nonetheless, in literature there can be found some studies in which CL is used as an excitation source in macro environments. Zhang et al. report a new photoelectrochemical DNA biosensor using chemiluminescence reaction as a light source to produce photocurrents^27^.

Moving to the micro scale, the current attempts to exploit CL potential as excitation optic source are very few and all concern the lab-on-a-paper field. Lan et al. cunningly embed the chemiluminescent reaction of Luminol in the chain of reactions useful for their purposes. Thus, the light deriving from the CL process plays an important role in the photoelectric chemical system presented in their work^28^. These types of application are strictly related to photoelectrochemical reactions and require that the luminescent reagents well match the other components of the chemical system. This may be far from the effective use of CL as stand-alone light source because it restricts its potential to a very narrow spectrum of applications.

One of the most relevant limits of this type of chemical reaction is its duration. Indeed, most known CL reactions exhibit a flash-type light emission. Of course, this intrinsic property affects all the possible applications of this principle as a stable light source. On a macroscopic scale, an interesting solution to obviate the lasting issue was to fabricate hydrogels containing chemiluminescent compounds. Thus, exploiting a slow-diffusion controlled catalytic mechanism, it has been possible to reach durable light emission^29^. Though this appreciable result, a long-lasting CL system (several hours) on a microfluidic platform (microscopic scale) is yet to be demonstrated.

Hence, we present here the fabrication and the characterization of a fully integrated and long-lasting optofluidic incoherent light source for LOC applications, by merging the microvolumes control capabilities given by microfluidics with the 3D geometry shaping freedom of the femtosecond micromachining technique.

## Results

**Figure 2:**
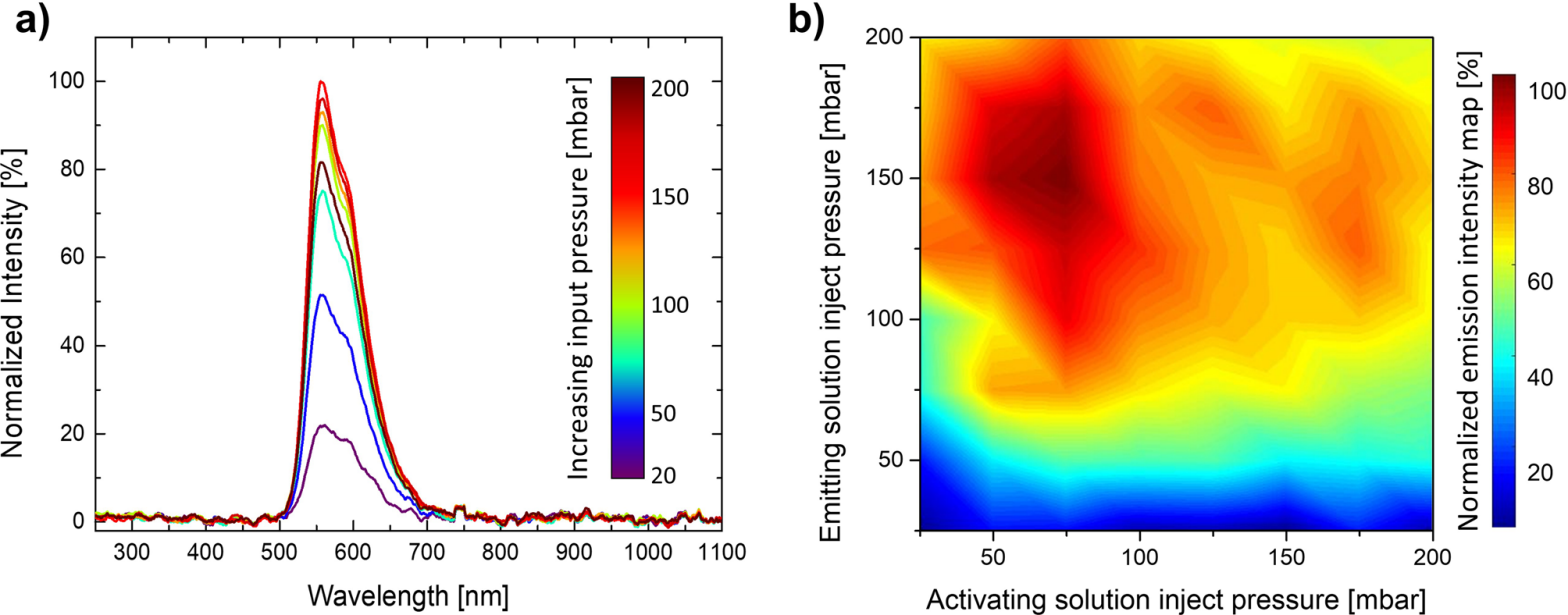
(**a**) Typical emission spectra obtained from the the triangular-shaped mixing chamber by acting on reagent injection flows. Spectra are normalized with respect to the maximum intensity of 200 nW. (**b**) Intensity versus reagents injection pressure map: the light emitted by the device is related to the chemical solution flow inside the mixing chamber. The emitting and the activating solution can react with different intensities as a function of the volume of reagents mixed inside the device.

Long lasting battery-free light sources can be fully integrated on microfluidic platforms by taking advantage of the well-known CL phenomenon of given organic-based solutions. The device exploits the following implementable working principle: the chemical energy stored in two reagents is transformed into electromagnetic radiation, normally in the VIS or N-IR spectrum, as a product of the reaction. The 3D designing freedom provided by the femtosecond laser micromachining technique gives the chance to exploit the fluidic nature of the chemiluminescent compounds to shape customizable emitting sources. The reaction happening inside the microfluidic chamber is controlled by the injection pressures of the input solutions. By varying the flow conditions of the reagents in the chamber (Fig. 2a), the tuning of the spectral behaviour in the emitted signal is achieved. The emitted spectra, using rubrene as dye, show a peak wavelength of 557 nm and a FHWM of around 80 nm. The peak changes both in intensity and in shape, shrinking, as a function of the flow.

The background noise emission or scattering from sample matrix components are almost absent: the spectrum baseline is practically flat. In fact, in the CL process the light is emitted only by a specific chemical reaction which involves the analyte. Indeed, light emission occurs exclusively when new reagents mix into the microfluidic chamber.

Numerical simulations have been carried out, showing a good match with the experimental mixing test with water-based dye solutions (see Supplementary Figs. S1, S2). From the numerical comparison of the three proposed geometries the mixing efficiency is slightly better for the circular shape. Though, experimentally both the circular and the square chambers were prone to air bubble-related issues. On the contrary, the acuminated shape provides the triangular chamber with a self-debubbling behaviour, making it preferable.

To better investigate the integrated light source behaviour, a description of the input–output relationship of the device has been carried out thanks to the pressure-intensity map—shown in Fig. 2b—obtained following the modalities shown in the characterization section. This suggests that the mixing is ruled by forced diffusion processes, as we have noticed by merging two dyed solutions and changing the input flow conditions (see Supplementary Fig. S1). The main consequence is that the photon emission is strictly regulated by the injection pressures but not always with a linear law. For greater flows, diffusion processes are too slow to let all the active molecules to meet and react. Indeed, the maximum intensity of the map shown in Fig. 2b is not linked to both the highest input pressures (see Supplementary Fig. S3). This phenomenon is further emphasized by a second factor: the concentration of the active compound in the emitting solution is higher than the one in the activating solution, as suggested by the patent^30^.

It is interesting to observe that, by using the same dye, the different geometries do not noticeably modify the behaviour of the emitted spectra—in fact, the radiation spectral proprieties are maintained for all those analysed. Conversely, taking in account the relation between light emission intensity and input pressures, the geometry plays a more relevant role resulting in different intensity maps (Supplementary Fig. S4).

**Figure 3:**
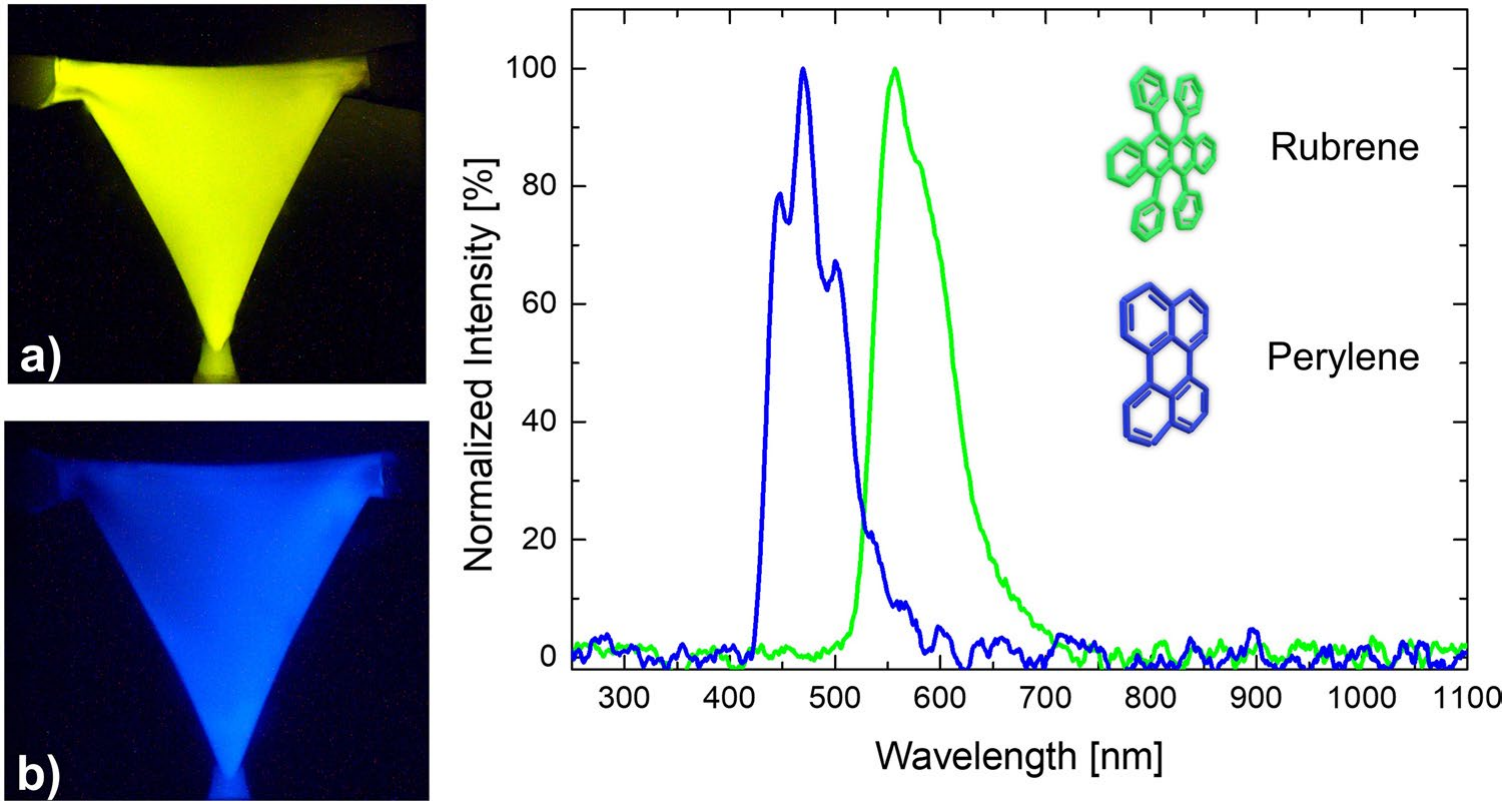
Spectral emission of the microfluidic mixing chamber. Light emitted by the rubrene-based solution (**a**) is centred in the green range*—*peak at 557 nm*—*of the visible spectrum, whereas perylene-based one (**b**) emits in the blue one*—*peak at 470 nm.

The design freedom of the light source can be further extended. Indeed, taking advantage from the solution-based CL process and the potential of the microfluidic platform, it is possible to easily manage the reagents within the device. Since the exploited CL is the indirect one, it is possible to tune the emission wavelength by simply injecting a new fluorophore keeping the same other solution components, as shown in Fig. 3. Consequently, the different reaction occurring inside the microfluidic chamber leads to a strong blue-shift in the emission spectrum, due to the injection of a perylene-based—instead of rubrene-based—solution. The emitted spectrum moves from the green range (Fig. 3a)—peak at 557 nm—to the blue area (Fig. 3b), namely at a 470 nm peak.

**Figure 4:**
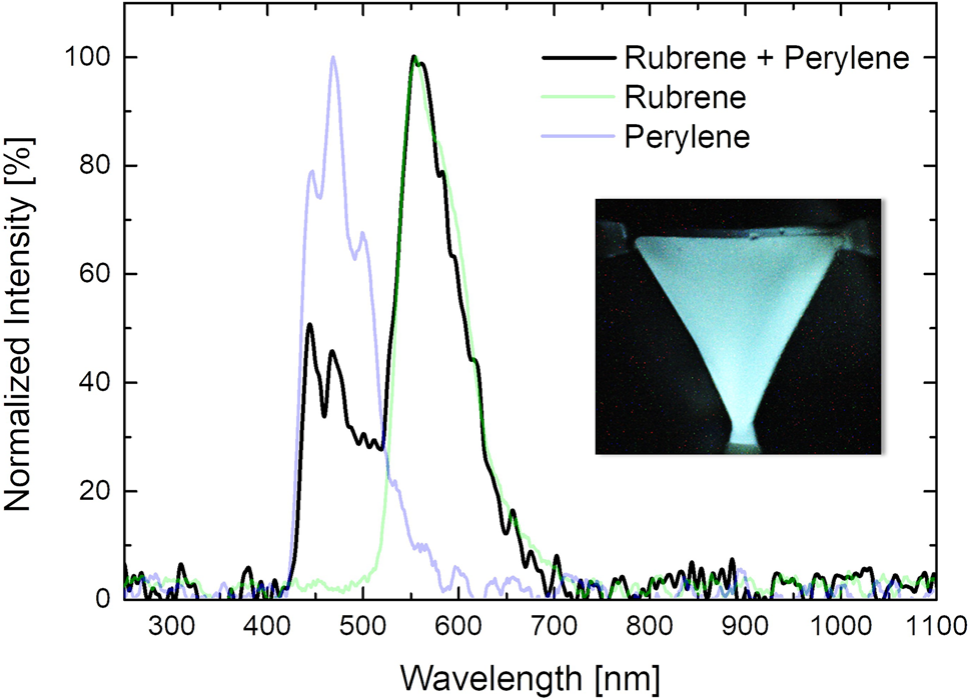
Spectral emission of a combination of Rubrene and Perylene in the mixing chamber. The emitted spectrum is the superimposition of the Rubrene and the Perylene emission spectra. The unbalance in the intensity of the peaks can be explained by the higher efficiency of the rubrene-based reaction and the likely quenching of blue light by rubrene molecules.

Figure 4 shows the "cool-white" emission resulting from an equally mixed solution of rubrene and perylene. It is interesting to note that the mixed spectrum is the superimposition of the two CL emitting compounds tested individually. Thus, the two different dye solutions do not react chemically with each other maintaining their basic functioning, while "superimpose" perfectly from an optic emission point of view. What is achieved is an adding between the individual colours. This could pave the way to a possible integrated RGB CL microfluidic source. By playing with reaction efficiencies, inlet pressures, combined absorptions and concentrations of the various compounds in the mixture, it is possible to modulate the different emission intensities of the single colour component. As a perspective, any colour temperature of white light in the chromaticity space may be possible. In the case shown in the Fig. 4, the intensity of the green component is greater than the blue mainly due to a twofold effect. Firstly, the rubrene reaction efficiency is higher. Then, a percentage of the blue emission is likely quenched by the rubrene molecules.

**Figure 5:**
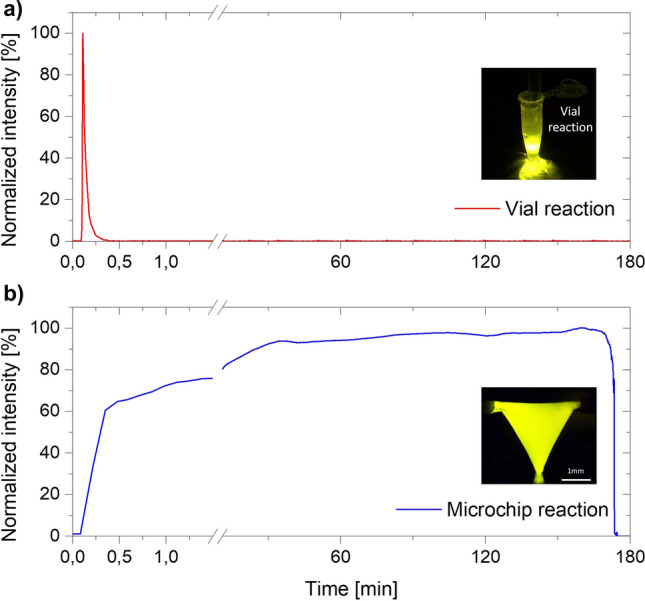
Light emission lasting time comparison. In both cases the reaction is achieved by mixing a fixed volume of reagents (0.7 mL each). (**a**) When the reagents are injected inside a vial, the consequent reaction lasts only for 30 s—i.e. the natural flash-type behaviour of CL reactions. (**b**) Instead by injecting in the microfluidic chamber the same volume of reagents it is possible to stretch the emission lasting time, elongating it to some hours (~ 3 h).

Another considerable strength in exploiting CL light sources within a microfluidic environment is the possibility to measure out the reagents to achieve a longer emission lasting time. Microfluidics gives the chance to accurately control small volumes—e.g. in the µL range for this device—and, thus, to manipulate the reaction, stretching it in time. Figure 5 compares the emission lasting time of a fixed volume of reagents—i.e. 0.7 mL both for the emitting and the activating solution—in two different cases. The first (Fig. 5a) is the chemiluminescent reaction on a macroscopic scale—i.e. the reagents are mixed in a vial. It is noticeable that, in this situation, the reaction follows its normal course, showing the typical flash-type (around 30 s) light emission. Conversely, when the chemiluminescent solutions are processed by the microfluidic mixing chamber, the emission can last much longer. In the results shown in Fig. 5b the input solutions are injected by a constant pressure of 200 mbar. The partitioning of the input volumes—achieved through the microfluidic circuit—leads to a ramp ascending more slowly to the emission peak. Once the plateau is reached, the light intensity is maintained quite constant until the reagents are finished. Thus, with these parameters, light can be constantly emitted up to about 3 h with an increase factor of over 350 times. By changing them—e.g. the reagents volumes ratio or decreasing the injection pressures—the lasting time can be further improved. Moreover, the microfluidic platform easily allows the reagent circulation: with constant new solution always available, the source can emit in "continuous" mode, virtually without time limitation.

## Discussion

We demonstrate the feasibility of a battery-free light source fully integrated on a microfluidic platform by taking advantage of a chemiluminescent process, where light can be emitted by merging two fluids. The presented device shows a high degree of flexibility either in the design—thanks to femtosecond laser micromachining—either in the operation modalities—exploiting the optofluidic advantages. Indeed, the microfluidic mixing chamber allows an easily reconfigurable emission of light, depending on the application. The wavelength tuning is simply achieved by changing or mixing the chemiluminescent compounds in the emitting solution.

Most known CL reactions exhibit a short-lasting time of the light emission which hampers their applications. Here, the idea is that, for a fixed amount of solution, a continuous and controlled flow of reagents in a microscopic chamber allows to obtain a constant luminous flux for an action time orders of magnitude longer than the emission time in a macroscopic environment. In the latter case, it is matter of fact that the radiation is more intense, but it is not constant. Conversely, in our device the emission intensity can be kept constant for long times, providing most lighting applications in LOC with an appealing tool. Rely on long-lasting fully integrated light emission is interesting for most of the applications, especially because it gives the opportunity to be independent from cumbersome external sources. This, together with the flexibility of the FLICE technique, can be exploited to design customized light sources for specific LOC applications.

The carried-out analyses, in particular 2D maps, show how the emission efficiency is linked to the constant pushing pressures of the reagents, once the chamber geometry has been established. Any method used to reach the chosen device working point—at constant injection pressure (or suction)—does not change the operation of the device. For instance, by generating a pneumatic positive (negative) pressure—through a micrometric mechanical (depression) valve or a syringe—at the outlet, the pressure gradient will push (extract) the reagents from the solution reservoirs without needing any battery. Thus, by embedding these general-purpose light sources in optofluidic platforms we push forward the path to the real portability of LOC devices.

## Methods

### Chip design and fabrication

**Figure 6:**
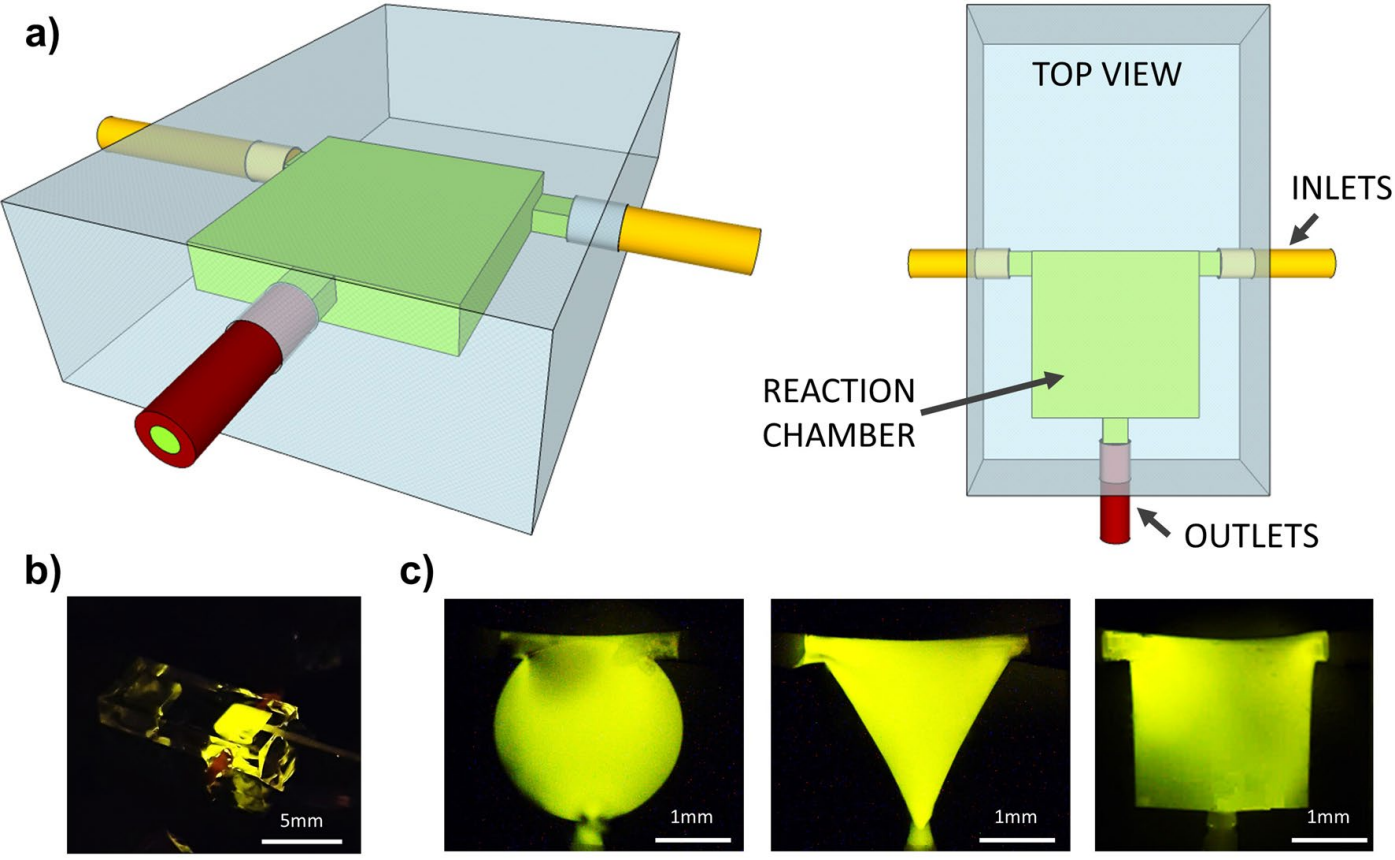
(**a**) Sketch of the chemiluminescent light source device. (**b**) Picture of the actual glass chip and zoom images (**c**) of different geometries obtained thanks to the 3D flexibility of femtosecond laser micromachining.

The devices created and tested in this study present the same fluidic circuit concept (Fig. 6a): two inlets for the reagents, a mixing chamber to promote the CL process and an outlet. The inlets are designed to merge in the mixing chamber facing each other, to guarantee an optimal contact area between the two reagents. In this way per each flow rate there still is emission and the reaction can be precisely controlled within the chamber.

Concerning the merging chambers, three basic geometries are fabricated and studied (Fig. 6c): triangular, circular and square. The mixing efficiency has been investigated through numerical simulations (COMSOL Multiphysics 5.3a). Due to the high complexity of the reagent compositions, the activating and the emitting solutions have been modelled in the simulations as two water based dyed solutions (see Supplementary Fig. S2).

The devices are fabricated taking advantage of the Femtosecond Laser Irradiation, followed by Chemical Etching (FLICE) technique. This maskless manufacturing method is based on two processes: direct 3D writing—by means of femtosecond laser pulses—of a fused silica substrate and subsequently chemical etching in a hydrofluoric (HF) acid water-based solution. In this way it is possible to obtain any hollow 3D microfluidic structure selectively where the substrate has been properly modified by the laser. Manufacturing the reaction chamber buried in the substrate is a critical point as it requires the removal of high volumes of material (over ~ 1 mm^3^) by a wet etching. Despite this, the FLICE fabrication process is able to well fulfil these compelling fabrication requirements. Moreover, thanks to this strategy, all the possible fluidic leakages can be avoided allowing to investigate the performance of the chemical light source in a wide range of injection pressures.

Our micromachining setup consists of an amplified Yb:KGW femtosecond laser system (Pharos, Light Conversion) with 230-fs pulse duration, 515-nm wavelength (frequency doubled), 500-kHz repetition rate focused with a 50 × —0.42 NA microscope objective (M Plan Apo SL50X Ultra-Long Working Distance Plan-Apochromat, Mitutoyo). Computer-controlled, 3-axis motion stages (ABL-1000, Aerotech) interfaced by CAD-based software (ScaBase, Altechna) with an integrated acousto-optic modulator are used to translate the sample relative to the laser irradiation desiderate patch. An average power (pulse energy) of 200 mW (400 nJ) and a scan speed of 1 mm/s are used to laser pattern the microfluidic devices. As reported in^31^, different beam polarisations are used to promote the etching process towards preferential directions to the detriment of the others. In this way, it is possible to obtain a selective control of the 3D geometry minimizing the etching time needed to make the chip. Concerning the chamber, its manufacture requires the removal of large volumes of internal material, therefore the writing methodology plays a decisive role in the success of the final device. The volume of interest is irradiated by using xy-overlapped planes, separated along the z-axis by 10 µm of unmodified material. For each xy plane, the area to be modified is filled with laser-irradiated lines with steps of 10 µm. The etching step is performed inserting the samples in a bath of 20% HF aqueous solution for about 7 h. The obtained chambers present a side—diameter for the circular shaped—of 2 mm and a thickness of 300 µm. To bring the solution inside the chambers, the devices are provided with three cylindrical accesses to house polymeric tubes (Fig. 6a) with an internal diameter of 50 µm—for the inlets—and of 150 µm—for the outlet.

## Materials

The chemiluminescent process exploited in this work is an indirect chemiluminescence reaction, therefore an intermediate step is needed (Fig. 1). The actual CL reaction, well described in^30^, follows the same rules and involves several compounds. The complex reaction can be simplified as the interaction of two main classes of solutions, namely “activating” and “emitting” solution. The activating solution is composed by sodium salicylate (2.9%) and hydrogen peroxide (97.1%) diluted in methanol while the emitting one by rubrene (7.8%)—for a yellow-greenish emission—or by perylene—for a blue emission peak—and bis(2,1,6 trichlorophenyl)oxalate (TCPO) (92.2%) in ethyl acetate. Percentages need to be intended as molar fractions.

Sodium salicylate acts as a catalyst in the reaction between TCPO and hydrogen peroxide, which leads to the production of a highly energetic intermediate. This compound immediately reacts with the dye that, accepting energy from the intermediate, moves towards an excited state. Then, the dynamics of relaxation from the excited state controls and strictly synchronizes the photon emission.

## Characterization

The device exploits an implementable working principle: to emit light the two chemical solutions need to meet inside the reaction chamber. To deep characterize the potentialities of this integrated light source, different experiments are carried out.

Of course, the injection pressure of the various chemiluminescent compounds into the microfluidic chip plays a very important role in the photonic performance of the light source. In order to make a complete, robust and systematic study of the photonic behaviour of the device, it has been useful—in a first place—to take advantage of a real-time control of the wide range of fluid dynamic parameters, such as pressure, flow rate and CL reaction time. For this reason, in this first CL light source characterization the reagents have been driven into the mixing chamber through a controlled pressure system by Elveflow (OB1MK3), that guarantees high resolution and stable control in the injection pressure regulation. However, we would like to underline that, once the optimal working point has been set for a given application (inlet pressure pair), any pressure control mechanism may be suitable for the correct device operation—e.g. mechanical (depression valve) and/or microfluidic (capillarity networks) systems. Following this idea, the electronic pressure control is not strictly needed.

Firstly, the emission intensity has been analysed as a function of the reagent inlet pressures. The obtained 2D-map shows (Fig. 2b) that the amount of light emitted is closely related to the capability of the chamber to facilitate the mixing and the meeting of all chemiluminescent reagent molecules. In this context, the pushing pressure and the chip geometry play an important role. The pressure range has been scanned between 25 to 200 mbar, with a 25 mbar step, for both the activating and the emitting solution. To ensure that the acquired spectrum is relative to the light signal emitted in a stationary fluidic regime, for each new combination of pressures it has been chosen to wait a time of about 50 s, enough to change at least 2 times the fluid volume contained in the chamber, before carrying out the acquisition. After this time, the spectrum has been collected from the device bottom by an optical fibre connected to a spectroscope (Spectra Wizard, SpectraWiz v5.33). The spectra have been acquired with an integration time of 2 s and no averaging among the scans. Light intensity has also been quantified by means of an integrating sphere (819C-SL-5.3-CAL2, Newport).

Then, the wavelength tuning capability of the device has been tested. There are numerous methods to tune the emission wavelength of an optofluidic device. One of the simplest and most significant is to change the dye. For example, by switching rubrene with perylene it is possible to observe a blue-shift of the emitted spectrum of 87 nm. However, microfluidics offers the great potential to handle several fluids at the same time (not just two) and therefore, with appropriate mixing stations it is possible to fine-tune the emission in a wide range of wavelengths.

In the end, we have tested the light emission lasting time. In this experimental analysis the injection reagent pressures are fixed to 200 mbar and a volume of 0.7 mL both for the activating and emitting solution is used. The emission spectra have been measured—1 spectrum every 8 s—from the beginning of the reaction until the end of the reagents—i.e. of the emission itself.

All data have been analysed with Origin 8.4 software.

## Supplementary information

Supplementary Information

## Data Availability

The datasets generated during and/or analysed in the current study are available from the corresponding author on reasonable request.

## References

[1] Yang RJ, Fu LM, Hou HH (2018). Review and perspectives on microfluidic flow cytometers. Sens. Actuators B Chem..

[2] Psaltis D, Quake SR, Changhuei Y (2006). Developing optofluidic technology through the fusion of microfluidics and optics. Nature.

[3] Monat C, Domachuk P, Eggleton BJ (2007). Integrated optofluidics: A new river of light. Nat. Photonics.

[4] Lee W, Yoon DK (2018). Optofluidic ring resonator laser with biocompatible liquid gain medium. Opto-Electron. Commun. Conf..

[5] Talik NA, Kar YB, Tukijan SNM, Wong CL (2017). Review on recent developments on fabrication techniques of distributed feedback (DFB) based organic lasers. J. Phys. Conf. Ser. Phys..

[6] Kong Y, Dai H, He X, Zheng Y, Chen X (2018). Reconfigurable RGB dye lasers based on the laminar flow control in an optofluidic chip. Opt. Lett..

[7] Simoni F, Bonfadini S, Spegni P, Lo Turco S, Lucchetta DE, Criante L (2016). Low threshold Fabry-Perot optofluidic resonator fabricated by femtosecond laser micromachining. Opt. Express.

[8] Criante L, Lucchetta DE, Vita F, Castagna R, Simoni F (2009). Distributed feedback all-organic microlaser based on holographic polymer dispersed liquid crystals. Appl. Phys. Lett..

[9] Williams G, Backhouse C, Aziz H (2014). Integration of organic light emitting diodes and organic photodetectors for lab-on-a-chip bio-detection systems. Electronics.

[10] Jahns S, Iwers AK, Balke J, Gerken M (2017). Organic optoelectronics for lab-on-chip fluorescence detection. Tech. Mess..

[11] Vezenov DV, Mayers BT, Wolfe DB, Whitesides GM (2005). Integrated fluorescent light source for optofluidic applications. Appl. Phys. Lett..

[12] Lim J-M, Kim S-H, Choi J-H, Yang S-M (2008). Fluorescent liquid-core/air-cladding waveguides towards integrated optofluidic light sources. Lab Chip.

[13] Pagliara S, Camposeo A, Polini A, Cingolani R, Pisignano D (2009). Electrospun light-emitting nanofibers as excitation source in microfluidic devices. Lab Chip.

[14] Garcia-Campana AM (2001). Chemiluminescence in Analytical Chemistry.

[15] Reschiglian P (2010). Chemiluminescence and Bioluminescence: Past, Present and Future.

[16] Pinzani, P., Messeri, G. & Pazzagli, M. Chemiluminescenza. *Caleidoscopio* **84** (1993).

[17] Timofeeva II, Vakh CS, Bulatov AV, Worsfold PJ (2018). Flow analysis with chemiluminescence detection: Recent advances and applications. Talanta.

[18] Amatatongchai M, Hofmann O, Nacapricha D, Chailapakul O, deMello AJ (2007). A microfluidic system for evaluation of antioxidant capacity based on a peroxyoxalate chemiluminescence assay. Anal. Bioanal. Chem..

[19] Far HRM, Torabi F, Danielsson B, Khayyami M (2005). ELISA on a microchip with a photodiode for detection of amphetamine in plasma and urine. J. Anal. Toxicol..

[20] Yakovleva J, Davidsson R, Bengtsson M, Laurell T, Emnéus J (2003). Microfluidic enzyme immunosensors with immobilised protein a and G using chemiluminescence detection. Biosens. Bioelectron..

[21] Huang X, Ren J (2005). On-line chemiluminescence detection for isoelectric focusing of heme proteins on microchips. Electrophoresis.

[22] Kloth K (2009). A regenerable immunochip for the rapid determination of 13 different antibiotics in raw milk. Analyst.

[23] He D, Zhang Z, Huang Y (2005). Chemiluminescence microflow injection analysis system on a chip for the determination of sulfite in food. Anal. Lett..

[24] Cheek BJ, Steel AB, Torres MP, Yu YY, Yang H (2001). Chemiluminescence detection for hybridization assays on the Flow-thru Chip, a three-dimensional microchannel biochip. Anal. Chem..

[25] Lee SH, Kim SW, Kang JY, Ahn CH (2008). A polymer lab-on-a-chip for reverse transcription (RT)-PCR based point-of-care clinical diagnostics. Lab Chip.

[26] Hu L, Xu S, Pan C, Zou H, Jiang G (2007). Preparation of a biochip on porous silicon and application for label-free detection of small molecule-protein interactions. Rapid Commun. Mass Spectrom..

[27] Zhang X, Zhao Y, Zhou H, Qu B (2011). A new strategy for photoelectrochemical DNA biosensor using chemiluminescence reaction as light source. Biosens. Bioelectron..

[28] Lan F (2017). Internal light source-driven photoelectrochemical 3D-rGO/cellulose device based on cascade DNA amplification strategy integrating target analog chain and DNA mimic enzyme. ACS Appl. Mater. Interfaces.

[29] Liu Y (2017). Firefly-mimicking intensive and long-lasting chemiluminescence hydrogels. Nat. Commun..

[30] Schleck, J. R., Keyko, G. J., Chopdekar, V. M. Two-component chemiluminescent composition. *Patent USA* **5597517** (1997).

[31] Taylor R, Hnatovsky C, Simova E (2008). Applications of femtosecond laser induced self-organized planar nanocracks inside fused silica glass. Laser Photon. Rev..

