# Supplementary material for: Battery-free fully integrated microfluidic light source for portable lab-on-a-chip applications
Source: Sci Rep. 2020 Jul 31;10:12910. doi: 10.1038/s41598-020-69581-z (PMC7395173; doi:10.1038/s41598-020-69581-z)
Supplement: Supplementary file 1 — Supplementary Information [file 41598_2020_69581_MOESM1_ESM.pdf]

BATTERY-FREE FULLY INTEGRATED MICROFLUIDIC LIGHT SOURCE FOR PORTABLE LAB-ON-A-CHIP APPLICATIONS

Filippo Storti[1,2], Silvio Bonfadini[1], and Luigino Criante[1,*]

[1]Center for Nano Science and Technology@PoliMi, Istituto Italiano di Tecnologia, via Pascoli 70/3, 20133 Milano, Italy
[2]Politecnico di Milano, Dipartimento di Fisica, Piazza Leonardo da Vinci 32, 20133, Milano, Italy
*Correspondence to (e-mail): luigino.criante@iit.it

Supporting Information

The evaluation of the mixing properties of the merging chambers is carried out by injecting two water-based food dyes and by scanning the working pressures of the device. The red and the blue dye are injected with equal pressures and the scanned range spans from 50 mbar to 200 mbar, with steps of 25 mbar.

Three bands of coloured solution emerge clearly from all the pictures. Closer to the injection ducts the dyes show their original colours (red and blue), while the central band is purple. This behaviour is typical of laminar flow where diffusion processes occur at the interface between the two fluids. By increasing the input pressures, the diffusion area is gradually confined in the central zone of the chamber. Since the chemiluminescent reaction happens just where the two solutions meet -i.e. the purple area-, this explains partially why the emitted light intensity doesn't present its peak at higher injecting pressures.

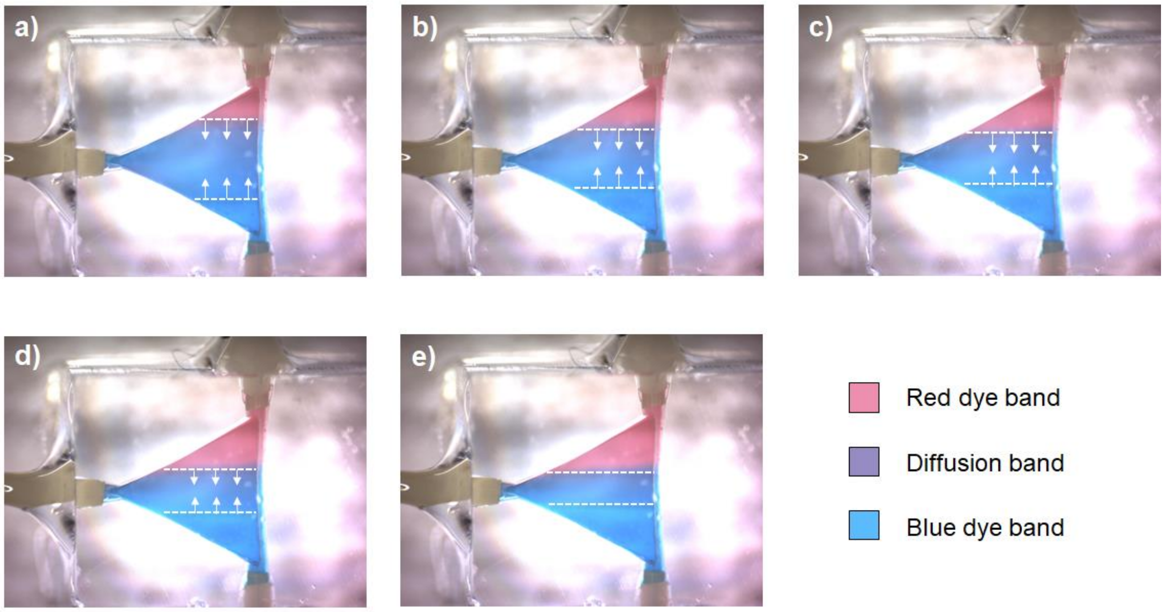

Supplementary Figure S1. Experimental forced diffusion flow assessing. Two water-based food dyes (red and blue) are injected with equal pressures in the chamber to evaluate its fluidic behaviour. The scanned pressures are (**a**) 50 mbar,(**b**) 75 mbar, (**c**) 100 mbar, (**d**) 150 mbar, and (**e**) 200 mbar. It's evident how three coloured bands are clearly defined inside the mixing chamber: the red, the blue, and the purple, where diffusion processes between the previous two bands occur.

Due to the too complex composition of the chemiluminescent solution and to the unknown parameters (such as the dynamic viscosity), numerical simulations have been carried out with some degree of approximation. The fluids mixing into the chamber have been modelled as water-based dye solutions: the red and blue colour in the simulation represent the un-mixed solution and complete mixing is achieved in the green areas.

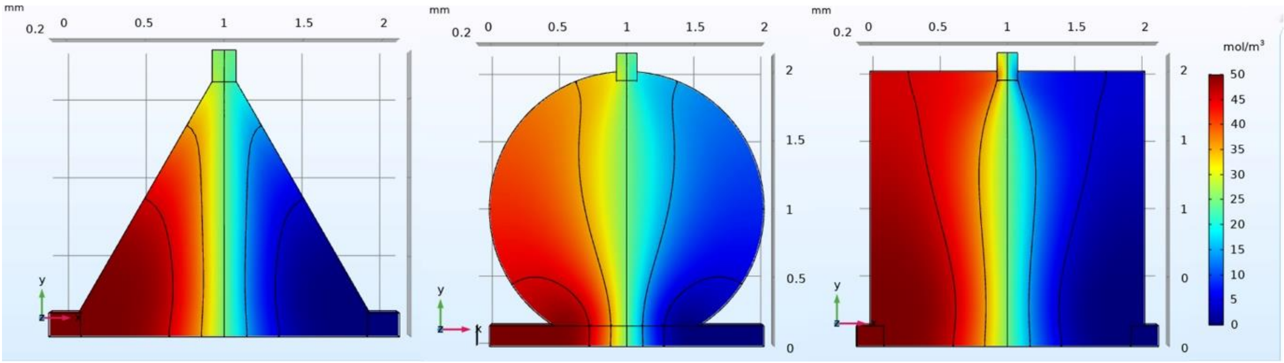

Supplementary Figure S2. Numerical diffusion simulations of the 3 mixing chamber geometries. Pure blue and red colors indicate the solutions not mixed, while the complete diffusion is achieved in the green areas. The injection pressures have been set at 50 mbar for both the inlets for all the geometries.

The microfluidic chambers showed a common behaviour: the light intensity is very low for low injection pressures, moderate for the highest pressures and the maximum is achieved for uneven pressure pairs - i.e. not on the diagonal of the map. As we can notice from Supplementary Fig. S3 a, although the flow is slow enough to let the diffusion processes take place, the number of molecules is sufficient to have just a low emission. Vice versa, when injection pressures are at the maximum, the number of molecules is increased but not all the molecules that potentially can react will do it. In fact, since flow velocity is high, they are flushed out of the chamber before they can emit- see the shadow area in Supplementary Fig. S3 c. Supplementary Figure S3 b shows the situation close-by the diagonal of the map: the light intensity has increased but still some mild shadow areas are present. The maximum light intensity is instead achieved for a couple of unbalanced pressures due to the CL mixture characteristics: in particular, the 2D map is influenced by the difference in the viscosity of the two solutions (activating and emitting), the different number of molecules that they carry, and the concentration gradient of the reagents.

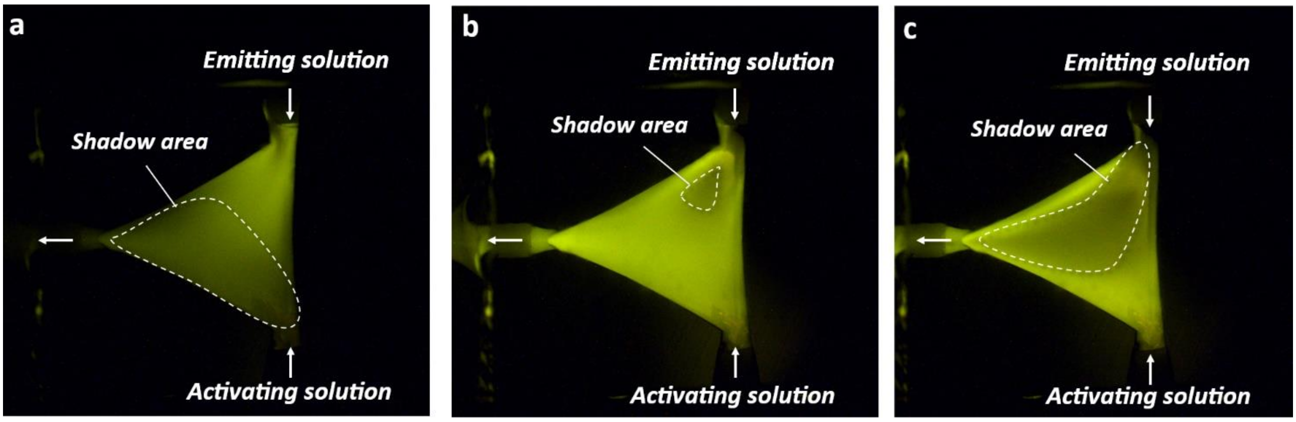

Supplementary Figure S3. Chemiluminescent chamber at 3 different experimental conditions. (**a**) Both emitting solution and activating solution are injected with a 25-mbar inlet pressure. The number of emitting molecules in the chamber is very low and thus the emitted light intensity itself. (**b**) Inlet pressures: 125 mbar (emitting solution) and 150 mbar (activating solution). (**c**) Inlet pressures: 200 mbar (both for emitting and activating solution).

Considering different device geometries – i.e. triangular (Fig. 2 b) and circular (Supplementary Fig. S4 a) - two main observations are relevant. Firstly, the light emission intensity maps are different, showing that geometry could play a role in the customization of the light source for specific purposes. On the other hand, the geometry change does not involve the light emission spectra, which remain the same for the chamber different shapes (Supplementary Fig. S4 b).

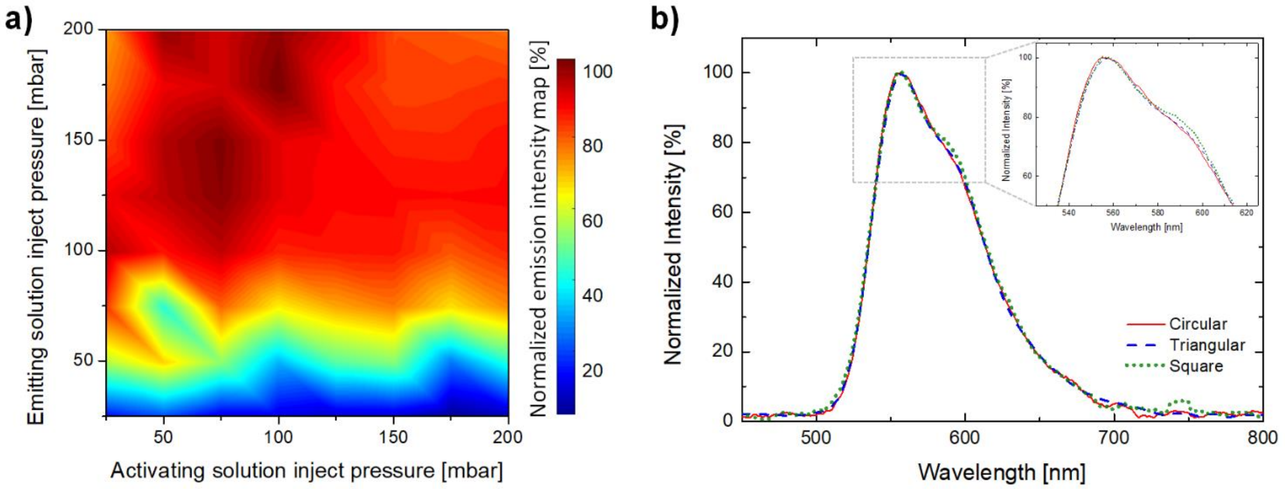

Supplementary Figure S4. Light emission comparison for different device geometries. (**a**) Light emission intensity maps for the circular-shaped mixing chamber. (**b**) Emitted spectra for triangular, circular, and square geometry: no appreciable difference can be noticed in the spectrum shape.
